# Exploration of autoantibody responses in canine diabetes using protein arrays

**DOI:** 10.1038/s41598-022-06599-5

**Published:** 2022-02-15

**Authors:** Allison L. O’Kell, Mahasish Shome, Ji Qiu, Stacy Williams, Yunro Chung, Joshua LaBaer, Mark A. Atkinson, Clive Wasserfall

**Affiliations:** 1 Department of Small Animal Clinical Sciences, College of Veterinary Medicine, The University of Florida, 2015 SW 16th Ave, Box 100116, Gainesville, FL 32608 USA; 2 The Virginia G. Piper Center for Personalized Diagnostics, Biodesign Institute, Arizona State University, Tempe, AZ USA; 3 College of Health Solutions, Arizona State University, Phoenix, AZ USA; 4 Department of Pathology, Immunology, and Laboratory Medicine, The University of Florida, Gainesville, FL USA

**Keywords:** Immunology, Endocrine system and metabolic diseases

## Abstract

Canine diabetes has been considered a potential model of human type 1 diabetes (T1D), however the detection of autoantibodies common in humans with T1D in affected dogs is inconsistent. The aim of this study was to compare autoantibody responses in diabetic and healthy control dogs using a novel nucleic acid programmable protein array (NAPPA) platform. We performed a cross-sectional study of autoantibody profiles of 30 diabetic and 30 healthy control dogs of various breeds. Seventeen hundred human proteins related to the pancreas or diabetes were displayed on NAPPA arrays and interrogated with canine sera. The median normalized intensity (MNI) for each protein was calculated, and results were compared between groups to identify candidate autoantibodies. At a specificity of 90%, six autoantibodies had sensitivity greater than 10% (range 13–20%) for distinguishing diabetic and control groups. A combination of three antibodies (anti-KANK2, anti-GLI1, anti-SUMO2) resulted in a sensitivity of 37% (95% confidence interval (CI) 0.17–0.67%) at 90% specificity and an area under the receiver operating characteristics curve of 0.66 (95% CI 0.52–0.80). While this study does not provide conclusive support for autoimmunity as an underlying cause of diabetes in dogs, future studies should consider the use of canine specific proteins in larger numbers of dogs of breeds at high risk for diabetes.

## Introduction

Diabetes mellitus (DM) is a common endocrine disorder in dogs with an increasing prevalence over time^1,2^. The disease is characterized by insulin deficiency, necessitates lifelong therapy with exogenous insulin, and in some ways is similar to type 1 diabetes (T1D) in humans^1^. Though much remains unknown regarding the pathogenesis of canine diabetes, contributing factors may include one or more of exocrine pancreatic disease, concurrent endocrinopathies such as hyperadrenocorticism leading to insulin resistance and secondary β cell dysfunction, or autoimmune destruction of the β cells^2,3^.

In human T1D, most cases are thought to result from β-cell directed autoimmunity leading to β-cell loss^4^. While autoantibodies are not themselves thought pathogenic in T1D (i.e., destructive for β-cells), they are commonly used as either diagnostic biomarkers of T1D or those at increased risk for the disease^5^. In terms of specific antigenic targets, they most commonly include antibodies targeting insulin, insulinoma associated protein 2 (IA2), glutamic acid decarboxylase 65 (GAD65), and zinc transporter 8 (ZNT8)^4^. Indeed, one or more of these autoantibodies are detected months to years before symptomatic disease ensues in nearly all subjects and more than 90% of patients are positive for at least one autoantibody at diagnosis^4,6^.

In dogs, studies evaluating for the presence of these autoantibodies have, unfortunately, reported inconsistent results, with 0–13% of dogs testing positive for GAD65 antibodies^7–9^, 0–10% of diabetic dogs testing positive for IA2 antibodies^8,9^, 3–12.5% of untreated diabetic dogs testing positive for insulin antibodies^10,11^, and 0% of diabetic dogs testing positive for ZnT8 antibodies^9^. Additionally, a small study evaluated autoantibodies against canine proinsulin, in which 53% of newly diagnosed diabetic dogs were positive^12^. Although islet cell cytoplasmic antibodies (ICA) have yet to be detected in naïve diabetic dogs^7,13^, approximately 50% of dogs in one study were noted as positive for serum anti-β-cell antibodies using purified islets utilizing a rat insulinoma cell line as an antigen^14^; a situation not unlike humans who are positive for ICA yet negative for other known autoantibodies^15^.

Beyond these commonly reported autoantibodies, multiple studies of humans with T1D have identified other novel autoantigens using a variety of techniques^16–19^. Two of these studies used an innovative Nucleic Acid Programmable Protein Array (NAPPA) platform to identify novel candidate autoantigens^18,19^. Unlike traditional protein microarrays that use purified proteins, NAPPA uses cDNA-encoding plasmids that are transcribed and translated in situ to create protein microarrays^18,20^. This method avoids some limitations of traditional purified protein arrays such as the time and cost of purifying multiple proteins as well as limited shelf stability^18^.

One possible reason for the lack of consistent evidence for autoimmunity in canine diabetes is that the relevant autoantibodies, and thus autoantigens, have not been identified, and a large proteome-scale search for autoantibodies in diabetic dogs has yet to been published. Given the similarities in genes between humans and dogs^21^, alongside the aforementioned quest to identify similarities between human T1D and canine diabetes, we elected to use a readily available human gene bank and the established NAPPA assay. Specifically, the objective of the study is to compare autoantibody responses in diabetic and healthy control dogs using a NAPPA platform.

## Materials and methods

### Dogs

Dogs were recruited from the client owned dog population from the University of Florida Small Animal Hospital. The study was approved by the Institutional Animal Care and Use Committee and the Veterinary Hospital Research Review Committee. The study was performed in accordance with associated guidelines and regulations. Owners provided informed consent prior to study enrollment. Dogs were enrolled between May of 2016 and November of 2019. Diabetes was diagnosed by the attending clinician based on the presence of hyperglycemia, glucosuria, and compatible clinical signs of diabetes (i.e., polyuria, polydipsia, weight loss). Diabetic dogs were included if they were a minimum of 3 kg body weight, at least 1 year of age, and, if female, were spayed prior the diagnosis of diabetes. Diabetic dogs that had a history of pancreatitis or hyperadrenocorticism were excluded. Healthy control dogs were included if they were a minimum of 3 kg body weight, at least 1 year of age, if a female were spayed, and received no other medications other than routine flea/tick/heartworm preventatives. Control dogs were deemed healthy based on a history and physical exam and a lack of clinical evidence of concurrent disease. Blood samples were collected via routine venipuncture into red top vacutainer tubes. Serum was separated routinely within 30 min of collection and frozen immediately at − 80 °C until analysis.

### Gene selection

Genes for NAPPA arrays were selected based on a literature search for human pancreatic genes, known genes important in human T1D screening, and candidate genes from an unpublished pilot study of diabetic dogs using NAPPA arrays. There were 1620 genes from literature search, 75 genes were known genes, and 5 genes from an unpublished pilot study for a total of 1700 genes (Supplementary Table 1).

### NAPPA arrays

NAPPA arrays were manufactured as previously described^22,23^. Briefly, bacterial clones having the genes of interest with a GST tag at the c-terminus, were obtained from the DNASU Plasmid Repository (DNASU.org). Plasmid DNA was purified using a mini-prep kit (Macherey–Nagel, #740499.50). DNA concentrations were then measured and normalized to 100 ng/µl for all 1700 genes. Silicon nanowell substrates were coated with (3-Aminopropyl) triethoxysilane (APTS) (Thermo Scientific, #80370) and then the plasmid DNA was printed using a piezo electric printer. At the time of usage, proteins were expressed from plasmid DNA using an in-vitro transcription and translation (IVTT) kit (Thermo Scientific, #88882). The printing quality of a batch was determined by expressing a random slide from the batch with the IVTT kit, followed by the detection of GST-tagged proteins with Mouse anti-GST antibody (Cell Signaling, #2624S) and Alexa 555 Goat anti-mouse IgG antibody (Invitrogen, #A-21422).

### Serological profiling on NAPPA

**Figure 1:**
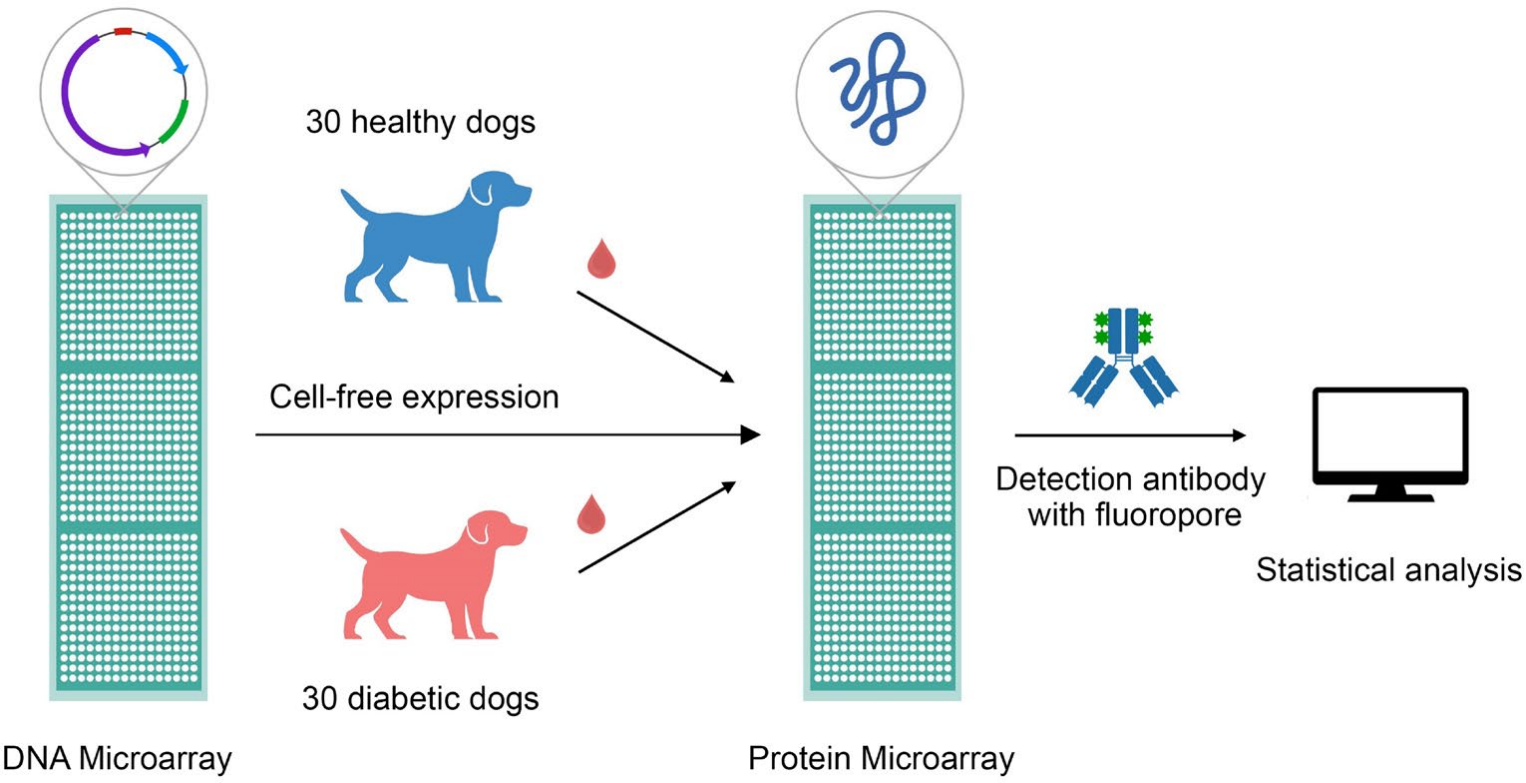
Flowchart explaining the study design. Initially, a DNA microarray is printed which is expressed using cell-free expression kit to make it a protein microarray. Sera from dogs were added followed by addition of detection antibodies.

Proteins were expressed using the IVTT kit and displayed on NAPPA. Dog serum samples diluted at 1:200 in PBST with 5% milk were added to the microarrays, followed by overnight rocking at 4 °C. After washing with PBST, dog autoantibodies were detected by 1:3000 diluted biotinylated anti-dog IgG (KPL, #16-19-06) followed by 1:2000 diluted Alexa 555 Streptavidin (Invitrogen, #S21381). Scanned microarray images were analyzed by the ArrayPro image analysis software. Antibody reactivity of each spot was normalized by division with the median spot intensity of each corresponding microarray. This normalized intensity value is denoted as Median Normalized Intensity (MNI). The study design is summarized in Fig. 1.

### Data and statistical analysis

Data recorded included age, breed, sex (and neutering status), body weight, duration of diabetes (if applicable), and concurrent medical conditions. The sample size was based on feasibility of enrollment for an exploratory study. Continuous data were tested for normality using the D’Agostino and Pearson test, and parametric or non-parametric tests used as indicated. Age and body weight were compared between diabetic and control groups using an unpaired T-test and Mann–Whitney *U* test, respectively, with sex distribution compared with a Chi Squared test.

We used the MNI values to analyze antibodies quantified on NAPPA. Seropositive proteins were defined as proteins whose antibodies having MNI values greater than an empirical cutoff of 1.5 on NAPPA. We compared the number of seropositive proteins between the diabetic and control groups using Wilcoxon rank-sum test.

Antibody sensitivity in the diabetic group at 90% specificity was calculated as follows. For each antibody, we calculated the threshold as the maximum between either the 90th percentile of MNI values in the control samples or the empirical seropositivity cutoff of 1.5. We then computed the corresponding sensitivity as the percentage of diabetic samples higher than the threshold. Antibodies with sensitivity greater than 10% were selected, and a subset of these were further selected as a panel of diabetic biomarkers using lasso logistic regression. Its discriminatory performance between diabetic and controls was evaluated by sensitivity at 90% specificity, the area under the receiver operating characteristics (ROC) curve (AUC), and their 95% confidence intervals (CI). GraphPad Prism (v8.0, San Diego, CA) and R (v4.1.0, Vienna, Austria) were used for the analyses.

Reporting for this study follows recommendations of the ARRIVE guidelines applicable to a veterinary clinical study.

## Results

Thirty diabetic dogs and thirty healthy control dogs were included in this study. The breed distribution for each group is shown in Table 1. The diabetic dog group had a mean age of 8.5 ± 3 years and were older than the control dog group, with a mean age of 6.7 ± 2.8 years (*P* = 0.015). Body weight was not different between the groups, with a median body weight of 10.2 kg (range, 5.5–33.1 kg) in the diabetic group and 10.6 kg (range, 4.4–45.1 kg) in the control group (*P* = 0.94). The diabetic group consisted of 18 males neutered and 12 female spayed dogs, which was not significantly different than the control group, which included 16 males neutered and 14 female spayed dogs (*P* = 0.60). Diabetic dogs had a median (range) duration of disease of 3 months (0–36 months).

**Table 1:** Breed distribution.

Diabetic group breed	Number of dogs	Control group breed	Number of dogs
Mixed	8	Mixed	7
Labrador Retriever	4	Labrador Retriever	5
Dachshund	3	Dachshund	3
Miniature Pinscher	2	Miniature Pinscher	1
Miniature Schnauzer	2	Miniature Schnauzer	2
Cairn Terrier	1	Golden Retriever	1
Toy Poodle	1	Miniature Poodle	1
Cavalier King Charles Spaniel	1	Cavalier King Charles Spaniel	1
Australian Shepherd	1	Australian Shepherd	1
Miniature Australian Shepherd	1	Miniature Australian Shepherd	1
Yorkshire Terrier	1	Flat Coated Retriever	1
Shih Tzu	1	Shih Tzu	2
Pomeranian	1	Pomeranian	1
Pembroke Welsh Corgi	1	Pembroke Welsh Corgi	1
Beagle	1	Beagle	1
Pug	1	Pug	1

**Figure 2:**
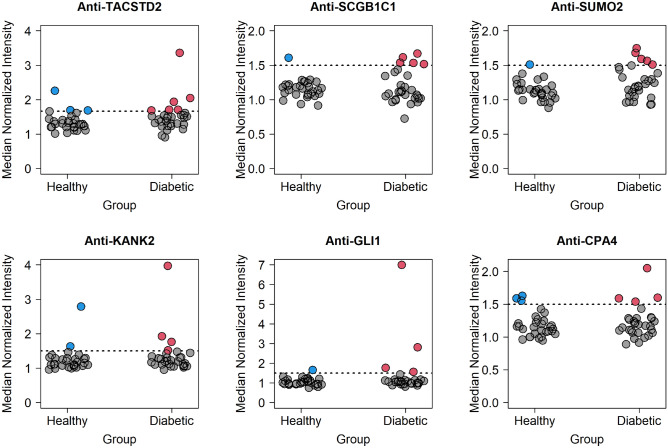
Reactivity of antibodies with sensitivities > 10% in diabetic and healthy dogs. Each dot represents an individual dog and the reactivity to the respective antibody. The horizontal dashed line represents the maximum between either the 90% percentile of the control samples or 1.5 and sensitivity is the proportion of red dots in the diabetic samples.

**Figure 3:**
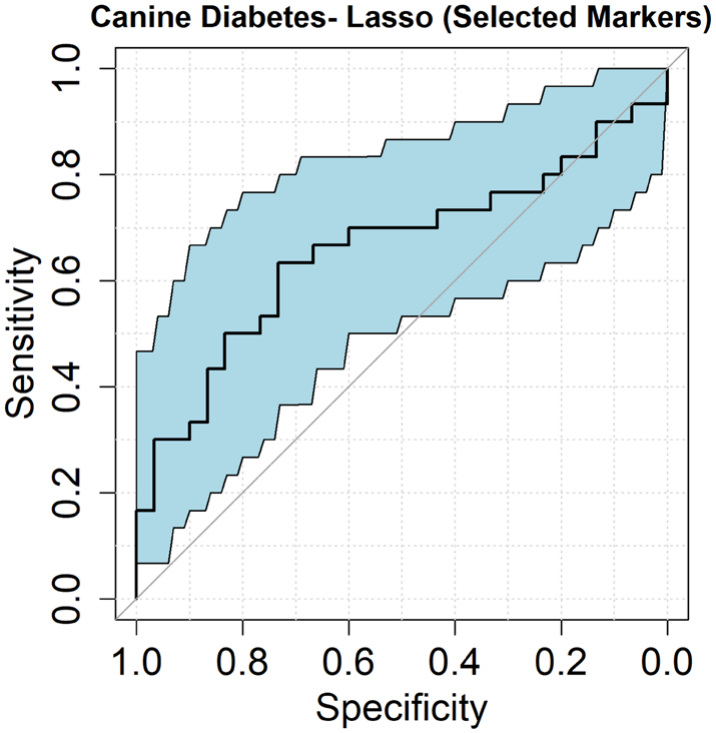
ROC curve. The antibody panel (Anti-KANK2, Anti-GLI1, Anti-SUMO2) was obtained from lasso logistic regression model with a sensitivity of 37% (95% CI 0.17–0.67%) at 90% specificity and an AUC of 0.66 (95% CI 0.52–0.80). The blue area represents the 95% CIs of sensitivities for each value of specificities, and the 45-degree straight line represents a useless biomarker having a sensitivity of 10% at 90% specificity and an AUC value of 0.5.

We evaluated the antibody profiles of these dogs against 1,700 human proteins relevant to diabetes and the pancreas. The number of autoantibodies with MNI values greater than 1.5 were 8.83 ± 9.37 and 9.93 ± 12.51 for the diabetic and control groups, respectively, a finding that was not significantly different (*P* = 0.74). At a specificity of 90%, six autoantibodies had sensitivity greater than 10%: anti-TACSTD2, anti-SCGB1C1, anti-SUMO2, anti-KANK2, anti-GLI1, and anti-CPA4 (Table 2 and Fig. 2). The proportion of positive results in each group are as follows: anti-TACSTD2 (6/30 diabetic, 3/30 control), anti-SCGB1C1 (5/30 diabetic, 1/30 control), anti-SUMO2 (5/30 diabetic, 1/30 control), anti-KANK2 (4/30 diabetic, 2/30 control), anti-GLI1 (4/30 diabetic, 1/30 control), and anti-CPA4 (4/30 diabetic, 3/30 control). With respect to multiple autoantibodies in diabetic dogs, 1 dog was positive for 5 autoantibodies, 1 was positive for 4 autoantibodies, 2 were positive for 3 autoantibodies, 2 were positive for 2 autoantibodies, and 9 dogs positive for a single autoantibody (Supplementary Fig. 1). Using lasso logistic regression, a subset of the aforementioned autoantibodies (anti-KANK2, anti-GLI1, anti-SUMO2) had a sensitivity of 37% (95% CI 0.17–0.67%) at 90% specificity and an AUC of 0.66 (95% CI 0.52–0.80) (Fig. 3).

**Table 2:** Proteins with sensitivity > 10% at a specificity of 90%.

Gene	Protein name	UniProt ID	Sensitivity (%)
TACSTD2	Tumor-associated calcium signal transducer 2	P09758	20
SCGB1C1	Secretoglobin family 1C member 1	Q8TD33	17
SUMO2	Small ubiquitin-related modifier 2	P61956	17
KANK2	KN motif and ankyrin repeat domain-containing protein 2	Q63ZY3	13
GLI1	Zinc finger protein GLI1	P08151	13
CPA4	Carboxypeptidase A4	Q9UI42	13

## Discussion

This is the first study, to the authors’ knowledge, to use a large proteomics-based approach to search for autoantibodies in canine diabetes. As noted, canine diabetes shares some features with human T1D, however the detection of the key autoantibodies found in the human disease has been inconsistent in dogs. Therefore, our goal was to broaden the scope of potential antigens screened in an attempt to find novel autoantibodies that may be important for the disease in dogs. The NAPPA arrays utilize a method that encourages proper folding of the translated proteins on the slide and therefore conformational epitopes should be available for antibodies to bind^24^. Using these arrays, displaying 1700 human proteins, we identified several candidate autoantibody/autoantigen combinations, however these have low sensitivities for distinguishing between diabetic and control groups.

Previous NAPPA array results in humans have identified novel minor type 1 diabetes associated antigens such as MTIF3, PPIL2, and MLHI^19^ in 7–24% of type 1 diabetic patients, along with small numbers of non-diabetic control patients, using a luciferase immunoprecipitation system^17^. Tetraspanin 7 autoantibodies are present in 35% of auto-antibody positive type 1 diabetes patients, but do not provide additional diagnostic value over the other established autoantibodies^16^. It has been suggested that these minor autoantigens may not be important for disease diagnosis, but may shed light into pathogenesis^17^.

Of the proteins with a sensitivity of > 10%, all of the genes have homologs or orthologs in dogs^25^, and none have been linked to diabetes in other species to the authors’ knowledge. However, several genes have been associated with other pancreatic disease, or fat and glucose metabolism. Both the CPA4 and TACSTD2 proteins are overexpressed in pancreatic carcinoma in humans and are associated with decreased survival^26,27^. Increased TACSTD2 gene expression is associated with increased fat mass in children^28^. In addition, SUMO2 expression has been reported as increased in rat mesangial cells exposed to high glucose conditions^29^, while CPA4 is a negative modulator of adipogenesis and insulin sensitivity^30^. The mechanisms leading to development of these autoantibodies require further study given the associations with the pancreas and metabolism in other species.

There are several potential reasons that we did not observe autoantibody candidates with higher sensitivities, including that our efforts did not screen all possible proteins. Additionally, although gene predictions have estimated that most of the almost 20,000 canine genes have human homologues^21^, the potential exists that some of the relevant canine proteins do not have human homologue or that the antigen binding sites on the canine antibodies do not recognize the epitopes on the human proteins.

Additionally, there is also growing evidence that canine diabetes, like human T1D, is a heterogenous disease. In dogs, strong breed predispositions suggest a genetic component contributes to disease risk^31^. Denyer et al. evaluated dog leukocyte antigen (DLA) (the canine equivalent to human leukocyte antigen) haplotypes in diabetic and control dogs (at least 20 in each group for each breed) in 12 different dog breeds^32^. They identified five dog breeds with DLA haplotypes associated with risk or protection, but other dog breeds, including 3 of the breeds at highest risk for diabetes, had no DLA associations with DM. This suggests that the disease may be heterogenous among breeds, especially with respect to immune related genes contributing to pathogenesis. Our study included small numbers of dogs of multiple dog breeds. Focusing screening on diabetic and control dogs in those dog breeds with DLA haplotype associations with diabetes risk may identify novel autoantibodies that are missed by testing a wide variety of breeds such as in the present study. Another limitation of the study is that dogs were evaluated at a single point in time, and it is possible that some diabetic dogs may have had autoantibodies earlier in the disease process or that some control dogs may have gone on to develop diabetes in the future. The younger age of the dogs in the control group could be a factor in our results, however differences in autoantibody reactivity among different ages of dogs has not been reported to the authors’ knowledge. Other studies performed with different techniques to evaluate for autoantibodies in canine serum have also reported some control dog reactivity when human assays were used^8,33^. The development of NAPPA arrays using canine specific genes/proteins is necessary to address this limitation and will afford future studies allowing for assessment, over time.

In conclusion, we identified six candidate novel autoantibodies in canine diabetes, however sensitivity to distinguish from non-diabetic control dogs was somewhat limited. This study does not provide strong support for the role of autoimmunity in disease pathogenesis in dogs using this set of genes and proteins; however, the small numbers of dogs of a variety of breeds are an important limitation. Future studies should focus on larger numbers of breeds considered high risk for diabetes using canine specific genes and proteins.

## Supplementary Information

Supplementary Figure 1.

Supplementary Table 1.

## Data Availability

The datasets generated during and/or analysed during the current study are available from the corresponding author on reasonable request.
