# Supplementary material for: Exploration of autoantibody responses in canine diabetes using protein arrays
Source: Sci Rep. 2022 Feb 15;12:2490. doi: 10.1038/s41598-022-06599-5 (PMC8847587; doi:10.1038/s41598-022-06599-5)
Supplement: Supplementary file 1 — Supplementary Figure 1. [file 41598_2022_6599_MOESM1_ESM.pdf]

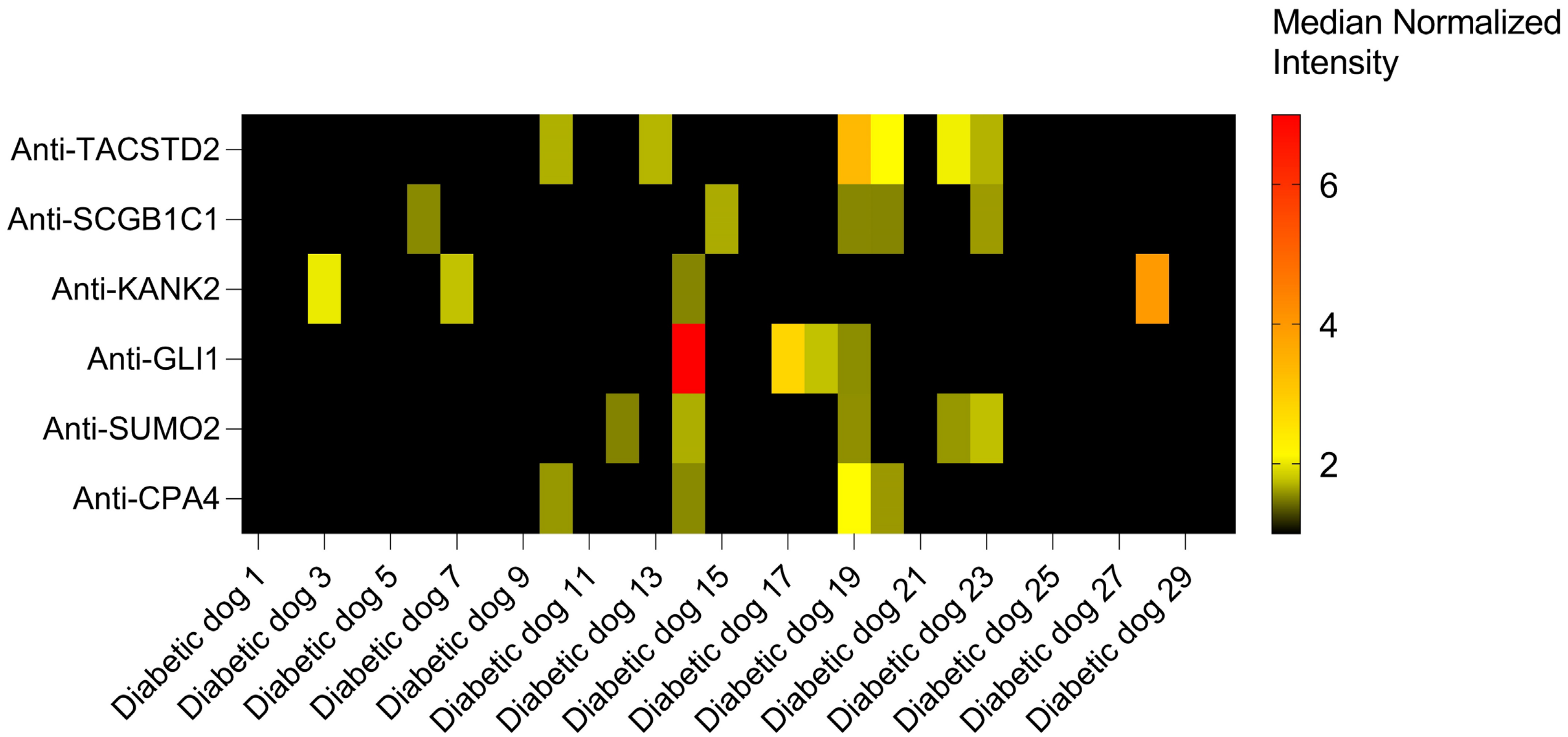

Supplementary Figure 1: Heat map of median normalized intensity (MNI) for diabetic dogs indicating seropositivity for each autoantibody. The cutoff for seropositivity was the maximum between either the 90th percentile of MNI values in the control samples or the empirical seropositivity cutoff of 1.5.
